# Quality of Life in Patients with End-Stage Renal Disease Undergoing Hemodialysis

**DOI:** 10.3390/jcm11061584

**Published:** 2022-03-13

**Authors:** Elżbieta Dembowska, Aleksandra Jaroń, Ewa Gabrysz-Trybek, Joanna Bladowska, Szymon Gacek, Grzegorz Trybek

**Affiliations:** 1 Department of Periodontology, Pomeranian Medical University in Szczecin, 70-111 Szczecin, Poland; elzbieta.dembowska@pum.edu.pl (E.D.); szgacek@gmail.com (S.G.); 2 Department of Oral Surgery, Pomeranian Medical University in Szczecin, 70-111 Szczecin, Poland; jaronola@gmail.com; 3 Department of Diagnostic Imaging and Interventional Radiology, Pomeranian Medical University, Unii Lubelskiej 1, 71-242 Szczecin, Poland; ewa_gabrysz@wp.pl; 4 Department of General and Interventional Radiology and Neuroradiology, Wroclaw Medical University, M. Curie-Skłodowskiej 68, 50-369 Wrocław, Poland; joanna.bladowska@umed.wroc.pl

**Keywords:** oral health, quality of life, oral-health-related quality of life, hemodialysis, end-stage renal disease, chronic renal disease

## Abstract

End-stage renal disease and hemodialysis therapy cause a number of changes, not only somatic but also psychosocial, including the patient’s perception and assessment of their quality of life. The literature describes predispositions to pathologies in the oral mucosa, craniofacial bones, teeth, and surrounding tissues in hemodialysis patients. This study aimed to determine the quality of life of hemodialysis patients in comparison with healthy subjects. The study group consisted of 200 subjects: the HD group (hemodialysis patients, *n* = 100) and the K group (control group, *n* = 100). General health and oral status were assessed using the following indices: plaque index, gingival index, probing depth, and clinical adhesion level. The WHOQOL-BREF survey was performed to determine both groups’ overall quality of life. The results showed lower values of assessed quality-of-life parameters in hemodialysis patients compared to the control group, especially in the somatic sphere. General diseases such as oral mycosis, osteoporosis, rheumatoid arthritis, and coronary-artery disease negatively impact the perceived quality of life. There are numerous indications for comprehensive psychological care of hemodialysis patients due to their poor psychosocial status.

## 1. Introduction

End-stage renal disease and hemodialysis therapy result in a number of changes, not only somatic but also psychosocial, such as the patient’s perception and assessment of their quality of life [1,2]. The success of hemodialysis therapy is the possibility of keeping the patient alive despite end-stage renal failure. The chronic nature of the treatment prompts us to cover the strictly biological aspect of the patients’ lives and psychosocial issues. Assessing patients’ quality of life allows the medical team to see the patient’s perspective holistically, not just paying attention to the patient’s diseases, and fosters physician–patient rapport building [1,3,4,5]. Sapilak et al. and Majkowicz et al. demonstrated the significant deterioration of patients’ quality of life due to dialysis treatment [1,2]. The subjective assessment of the quality of life of those on hemodialysis is one-third worse than in a comparable group not treated with hemodialysis [2]. The increased risk of complications, morbidity, and mortality in patients on hemodialysis is associated with decreased quality of life. As patients are limited in these activities of daily living, both their physical and psychological quality of life is reduced [6].

Moreover, the authors noted an analogy between hemodialysis patients’ worse quality of life and oncology patients compared to peritoneal-dialysis patients and the control group. In this study [1], the Hospital Anxiety and Depression Scale (HADS) and the Aggression Scale were used to compare the intensity of negative emotions in the study groups. Hemodialysis patients showed the highest level of aggression among the four studied groups of patients: oncology patients, peritoneal-dialysis patients, hemodialysis patients, and healthy controls. Anxiety levels were also higher among hemodialysis patients than peritoneal-dialysis patients and healthy controls. The level of depression in the group of hemodialysis patients was comparable to that of oncology patients and significantly higher than that of peritoneal-dialysis patients and controls. All the relationships described above were defined as statistically significant (*p* < 0.05). Using The European Organization for Research and Treatment of Cancer Quality of Life Questionnaire (EORTC-QOLQ-C30) quality-of-life scale, the authors noted a particularly negative assessment of social function in the group of hemodialysis patients compared to other study groups [1]. Majkowicz et al. also observed a difference between the nature of the psychological burden of hemodialysis patients, resulting from the inconvenience of the applied therapy, and resulting in irritability and aggression of greater intensity than, for example, in the group of oncology patients. The prevalence of depression in hemodialysis patients is about 20%, compared to 2–10% in the general population [7]. According to the FDI, “Oral health is multifaceted and includes the ability to speak, smile, smell, taste, touch, chew, swallow and convey a range of emotions through facial expressions with confidence and without pain, discomfort or disease of the craniofacial complex” [8]. As assessed by the oral-cavity condition, oral health is an essential part of a patient’s general health and is thus an integral component of quality of life. Oral conditions can have a significant impact on oral-health-related quality of life [9,10]. Oral diseases can affect physical, social, or psychological problems. As kidney disease and hemodialysis can affect patients’ oral health, both factors can affect the quality of life. The condition of the oral cavity, especially the occurrence of lesions and their pathology and healing, is influenced by the patient’s chronic diseases, such as renal disease [11]. There is also a correlation between psychological factors and the severity of periodontitis [12]. In the literature, a predisposition to the development of pathology in the oral mucosa, craniofacial bones, teeth, and surrounding tissues are described in hemodialysis patients. Chronic kidney disease also contributes to salivary-gland dysfunction and olfactory and taste receptors [13,14,15]. Skiba et al. reported a significant association of quality of life with oral-health status, so it can be inferred that the poor oral health of patients may affect their quality-of-life perception [16]. It has also been reported that bone disorders in chronic kidney diseases may affect patients’ quality-of-life assessment [17]. Patients’ perceptions of quality of life may also positively or negatively modify treatment outcomes [18]. Quality of life can be assessed using a variety of validated questionnaires [19]. Unfortunately, only few studies in the literature have assessed the quality of life in hemodialysis patients with respect to oral status [20]. Therefore, an attempt was made to determine the quality of life in patients with renal failure undergoing hemodialysis, in whom the oral condition was also evaluated. This study aimed to determine the quality of life of hemodialysis patients compared to healthy subjects. The following null hypothesis was defined: Chronic renal failure and oral status do not affect the quality of life.

## 2. Materials and Methods

The study was conducted in a group of hemodialyzed patients with chronic kidney disease. The consent of the Bioethics Committee of the Medical University was obtained (no. K0012/45/11. Individuals that qualified for the study gave informed consent for their participation and were informed in detail about its purpose and course.

The study group consisted of 200 subjects: the HD group (hemodialyzed patients, *n* = 100) and the K group (control group, *n* = 100). The control group was selected to correctly match the study group in terms of age and gender. Among the hemodialysis patients, the mean age was 55 years (±16.43), of which 42% (*n* = 42) were female, and 58% (*n* = 58) were male. The control group, which had a mean age of 52 years (±15.46), consisted of 43% (*n* = 43) women and 57% (*n* = 57) men (Table 1).

The following inclusion criteria were adopted for the study group:

- Duration of dialysis of at least three months
- The presence of end-stage chronic renal failure
- Informed consent to participate in the study

The exclusion criteria included:

- Taking immunosuppressive or cytotoxic drugs currently or in the past
- Disseminated malignancy
- Antibiotic therapy at the time of the study or within the past three months
- An acute infectious disease in the oral cavity, pharynx, and salivary glands at the time of the study

### 2.1. Clinical Examination and Anamnesis

The physical examination consisted of a general medical and dental history, including patients’ hygiene behaviors and the World Health Organization WHOQOL-BREF quality-of-life questionnaire [21,22].

The general medical history included information on general health status, duration of dialysis therapy, and concomitant diseases. Patients’ weight and height data were also obtained from the interview to allow assessment of body-mass index (BMI) [23].

The quality-of-life study was conducted using the WHOQOL-BREF questionnaire. The authors used the WHOQOL-BREF to analyze all aspects of quality of life in dialysis patients. The WHOQOL-BREF is a comprehensive, state-of-the-art tool for examining a patient’s quality of life. The WHOQOL-BREF has been translated into 50 languages and has been successfully used in patient-quality-of-life surveys worldwide [21]. The WHOQOL-BREF questions allow us to determine patients’ perceptions of quality of life in a cultural context, while also taking into account the patient’s value system and their expectations from life. The data obtained are insightful, measurable, and comparable in the context of literature studying patients’ quality of life worldwide [21].

The questionnaire consists of 26 questions covering four domains of life: somatic, social, psychological, and environmental. The questions in the WHOQOL-BREF specifically address relevant aspects of the described somatic, social, psychological, and environmental domains of life in a standardized manner. The somatic domain (D1) includes pain and discomfort, energy and fatigue levels, rest and sleep, treatment dependence, mobility, daily activities, and the ability to undertake work responsibilities. The psychological domain (D2) consists of positive and negative emotions, self-esteem, cognitive processes, body image, and the realm of spirituality. The social domain (D3) is defined by assessing personal relationships, sex, and real support from loved ones. The environmental domain (D4) includes financial resources, access to knowledge and skills, entertainment and recreation, residential environment, access to medical care, level of safety, and access to transportation.

### 2.2. Physical Examination

The patients’ oral hygiene was assessed using the plaque index (PI) according to Silness and Löe [24]. Periodontal status was also evaluated using the gingival index (GI) according to Löe and Silness [25].

In addition, the study assessed detailed periodontal status in the form of:

- Measurement of periodontal pocket depth (probing depth—PD),
- Clinical attachment level (CAL).

Periodontitis was classified using the periodontitis division according to Page and Eke [26].

The condition of the oral mucosa was also assessed with the detailed notation of the type of lesions, nature of complaints, and their location.

### 2.3. Statistical Analysis

The Kolmogorov–Smirnov test was used to determine the normality of the distribution of the variables. Characterization of variables was performed using means, standard deviations, and outliers. The Student’s *t*-test and Mann–Whitney test were used to examine the differences between the study groups (HD, K). Pearson’s test and Fisher’s exact test were used to reflect the relationships between discontinuous variables. Analysis of variance (ANOVA) or the Kruskal–Wallis test were also used to describe the groups.

Using frequency and number of occurrences, discontinuous variables were described, between which relationships were characterized using Pearson’s χ2 (chi-square) test.

Spearman’s rank correlation was referenced to assess the correlation between discontinuous variables (nominal and ordinal) and continuous variables, illustrated by the correlation coefficient r and probability *p*.

The questionnaires used were checked for reliability (agreement of all items in the sum scale). The α-Cronbach’s reliability coefficient was calculated.

Statistically significant differences presented a confidence level of *p* < 0.05. Statistical analysis was performed using the STATA 11 program (license number 30110532736).

## 3. Results

### Comparison of Quality-of-Life Levels in the Study Groups

Quality of life was assessed using the WHOQOL-BREF questionnaire. The α-Cronbach coefficient determined the quality-of-life-measurement method’s adequacy. The coefficient value was 0.94 for the hemodialysis patients (HD) and control subjects (K). Then, according to the design of the WHOQOL-BREF research tool, quality of life was summarized in terms of overall quality of life, satisfaction with health (Table 2), and four domains: somatic, psychological, social, and environmental (Table 3). The WHOQOL-BREF questionnaire values of overall quality of life range from 1 (“very poor quality of life”) to 5 (“very good quality of life”). In the study group (HD), the overall quality of life (overall quality of life)was assessed by hemodialysis patients at a mean level of 3.30 (±0.99), in the control group (K), the subjects perceived the average overall quality of life as being 4.02 (±0.78). The difference in the study groups is statistically significant (*p* = 0.000). Similarly, health satisfaction (health satisfaction, general health) [14], described by the WHOQOL-BREF questionnaire, is rated in a range of values from 1 to 5 (“very bad” to “very good”). In the HD group, the mean value of health satisfaction was 2.43 (±0.99). The differences described are statistically significant (*p* = 0.000).

The quality-of-life domains examined with the WHOQOL-BREF were also compared, with mean values on a 0–100 scale between the two study groups showing statistically significant differences (*p* = 0.000) (Table 3). The somatic (physical) domain in the study group (HD) obtained the lowest mean value of 56 (±23) out of all the evaluated aspects of quality of life, while in the control group (K) the value was 63 (±12). The psychological domain obtained the second lowest value of 61 (±16) for the HD group and 72 (±10) for the K group. The social domain obtained a value of 65 (±42) for the HD group and 87 (±36) for the K group.

The correlations between the quality-of-life assessment depicted by individual domains of WHOQOL-BREF and age, gender, and education level were also examined (Table 4). In the hemodialysis (HD) group, inverse correlations were noted between the age of the subjects and the perception of quality of life (R = −0.22, *p* = 0.0305) and all WHOQOL-BREF domains. These associations showed statistical significance (*p* < 0.05).

In the control group (K), a statistically significant relationship was noted only between the age of the subjects and the psychological and social domains in the form of the inverse proportionality of both characteristics (*p* < 0.05). There was also a correlation between the gender of the subjects in the HD group and the perception of quality of life and health and quality-of-life domains. In the HD group, the male gender was predisposed to lower values of the discussed aspects of quality of life (*p* < 0.05). No such relationship was found in the control group (K). There was no statistically significant correlation between the educational level of the HD and K groups and the discussed aspects of quality of life (*p* > 0.05).

The effect of stress on the subjects’ quality of life was also determined (Table 5). An inversely proportional relationship was found between stress and the level of perception of quality of life and the psychological domain in the control group (K).

A correlation analysis was also performed between the parameters of periodontal status and oral hygiene and the studied quality-of-life domains (Table 6). In the HD group, statistically significant correlations were noted between PD values and the patients’ social domain of life. As the mean PD values increased, the social domain of life assessment in the HD group decreased (*p* = 0.0018). Similar correlations were observed between the CAL and PI values according to Sillness and Löe and the HD group’s social and psychological domains of quality of life (*p* < 0.05). Higher gingival-index values were associated with lower health-perception scores in the HD group (*p* = 0.032). In this aspect, no statistically significant relationships were found among the study participants in the control group (K).

The effect of the number of retained teeth and tooth loss due to periodontal disease on patients’ quality of life was also evaluated (Table 7). In the study group (HD), there was a directly proportional relationship between the number of teeth retained in the oral cavity and the psychological and social domains of quality of life (*p* < 0.05). Tooth loss due to periodontal disease was associated in the HD group with lower psychological and social domains (*p* < 0.05). In the control group, a directly proportional relationship was found between the number of retained teeth and the social domain of quality of life (*p* < 0.05).

We also examined the correlations between the presence of comorbidities and quality of life in hemodialysis (HD) patients (Table 8). The relatively strongest correlation was found between the presence of osteoporosis and the social domain of life (R −0.36, *p* = 0.0002). This was followed by a relatively strong correlation between coronary-artery disease and the psychological domain of quality of life (R −0.35, *p* = 0.0003). The coexistence of coronary-artery disease in hemodialysis patients also contributed to lower values of the perception of quality of life and a weaker assessment of the somatic domain of quality of life (*p* < 0.05). A negative effect of diabetes on the psychological and social domains of quality of life was also noted (*p* < 0.05). The presence of oral mucosal candidiasis also negatively affected the perception of quality of life of hemodialysis (HD) patients (R −0.29, *p* = 0.0037).

There was also a negative association between food restriction due to periodontal disease and the somatic domain of quality of life (R −0.21, *p* = 0.032) (Table 9).

## 4. Discussion

Due to the burdensome disease and overwhelming therapy, hemodialysis patients show lower dynamics of life activity. The negative dimension of their health and person is characteristic of this group of patients [27]. It is believed that hemodialysis patients are particularly vulnerable to developing psychiatric disorders, including depression, neurosis, and pathological anxiety [7]. Together with somatic causes, these phenomena negatively affect the feelings related to the quality of life and constitute the specificity of this group of patients [28].

The questionnaire used in this study allowed us to measurably demonstrate the patients’ quality of life. At the outset, the reliability and adequacy of the data obtained using the WHOQOL-BREF questionnaire for the population of hemodialysis patients in the study population were confirmed (α-Cronbach coefficient 0.94). It should be noted that the WHOQOL-BREF is also a reliable and sensitive tool for assessing the quality of life of hemodialysis patients in other regions of the world [29]. The WHOQOL-BREF questionnaire also shows high adequacy in general-population studies (α-Cronbach coefficient 0.91) [30].

The perception of the quality of life of hemodialysis patients in the WHOQOL questionnaire used in this study was assessed at a mean level of 3.30 (±0.99), compared to 4.02 (±0.78) in the control group (K) (*p* = 0.000). The perception of the health of hemodialysis patients reached a mean value of 2.43 (±0.99), while those in the control group reached 4.08 (±0.99). From the above data, it can be concluded that there was a worse perception of quality of life and health in hemodialysis patients than in the control group.

Additionally, in the study of Sathvik et al. [5], a statistically significant reduction in quality of life was found in hemodialysis patients compared to healthy subjects (*p* < 0.05).

The reduction in the perception of quality of life is evident in the four domains of quality of life studied. In the group of hemodialysis patients, it was observed that the somatic (physical) domain obtained the lowest mean value (56 ± 23) of all the evaluated aspects of quality of life. In the general population, this value was higher (63 ± 12). The psychological domain in hemodialysis patients was rated at an average of 61 (±16) compared to 72 (±10) in the general population. In the author’s study, up to 6% of hemodialysis patients were reported to have serious psychological disorders, including depression and neurasthenia. One factor compounding this problem may be that up to 75% of hemodialysis patients were found to have chronic stress compared to 4% of the control group (*p* = 0.000).

The quantity and quality of social interactions also declined in the hemodialysis-patient group. In the social domain, hemodialysis patients scored a mean of 65 (±42), while the general population scored 87 (±36). The environmental domain in hemodialysis patients was 67 (±18), and in the general population, 82 (±14). In contrast, Sathvik et al. [5] observed the highest quality-of-life-assessment values in hemodialysis patients in the environmental domain with a mean of 60.59 (±11.73). Additionally, in the social domain a mean of 53.93 (±16.91), in the psychological domain a mean of 40.92 (±18.66), and in the physical domain a mean of 38.81 (±18.36). The authors [5] also compared the level of the quality of life of hemodialysis patients and kidney-transplant patients, and observed lower values in the four examined domains in HD patients compared to kidney-transplant patients (*p* < 0.05). In a study by Sreejith et al. [31], they observed the following quality-of-life domains among hemodialysis patients: environmental domain with a mean of 55.93 (±15.64), social domain with a mean of 55.43 (±19.92), psychological domain with a mean 49.21 (±15.83), and physical domain with a mean 44.05 (±14.02). Perlman et al. [32] described that the quality of life of hemodialysis patients is worse than those with earlier stages of chronic renal failure and those in the general population. In the study by Hawthorne et al. [30] for the general population, the values of individual domains were: for environmental a mean of 0.79, for social a mean of 0.68, for psychological a mean of 0.78, and for physical a mean of 0.87. Majkowicz et al. [1] observed that hemodialysis patients scored unfavorably in many quality-of-life domains compared to peritoneal-dialysis patients and healthy controls. Segelnick et al. [33] noted the importance of visiting a periodontal specialist for diagnosis and oral decontamination in the context of planned kidney transplantation and the associated immunosuppression. Bayraktar et al. [34], based on their study results, suggested the necessity of regular dental check-ups with repeated instruction in proper oral hygiene. A program of regular dental visits should be established and directly recommended to patients by dialysis centers in order to improve the general health and quality of life of these patients. In conclusion, based on the results obtained in this study and the literature, periodontal and general dental diagnostics and treatment are indispensable elements of prophylactic, therapeutic, and interdisciplinary management among hemodialysis patients with end-stage renal disease [34]. The systematically increasing number of patients requiring chronic hemodialysis draws attention to the problems of comprehensive health care in this group of patients. The improvement of dialysis techniques favors the prolongation of life and duration of therapy. This situation sheds new light on the health problems of this population. The long treatment period imposes the necessity of the full control of factors that are likely to interfere with its course. The oral health of patients, also expressed by the condition of the periodontium and mucosa, has significant potential to modify patients’ overall health. At the same time, behavioral, psychological, and social factors can create an atmosphere that promotes or even worsens a patient’s condition. Their subtle interactions with the body’s somatic harmony, which is disrupted by deterioration, determine patients’ quality of life. At the same time, patients’ quality of life is a barometer of the general condition of a person undergoing long-term treatment and struggling with the complications of the disease. Thus, the oral-health status of dialysis patients contributes to the deterioration of their outlook on life, i.e., it affects their quality of life. One treatment in this specific group of patients should be consistent prophylaxis and diagnosis of possible causes of the deterioration of the general health of these patients, including oral health [35]. Our study has some limitations. Page and Eke’s classification of appendage diseases was used in this study. In future studies, we plan to use the latest classification. In addition, convenience sampling was used in our study due to the difficulty in collecting the study group. All patients available at the specified time who met the inclusion and exclusion criteria were included in the study.

## 5. Conclusions

Based on the obtained results, the following was found: lower values of assessed quality-of-life parameters in hemodialysis patients compared to the control group, especially in the somatic domain; general diseases such as oral mycosis, osteoporosis, rheumatoid arthritis, and coronary-artery disease negatively impact perceived quality of life; numerous indications for the implementation of comprehensive psychological care of hemodialysis patients due to their poor psychosocial condition.

## Figures and Tables

**Table 1:** Summary of mean and extreme values of age in both study groups.

	Study Group (HD)		Control Group (K)		*p*
Mean age	55.18 (±16.43)		52.58 (±15.46)		0.2207
Minimum age	19.00		18.00		
Maximum age	85.00		83.00		
Q25	43.00		40.50		
Me	55.00		54.00		
Q75	67.00		64.50		
Mean age by gender	Female	Male	Female	Male	
	54.88	55.40	52.67	52.50	

**Table 2:** Comparison of overall quality of life and health satisfaction assessed by the WHOQOL-BREF quality-of-life questionnaire in the study groups (HD, K).

The Domains of Quality of Life Assessed by the WHOQOL-BREF Questionnaire		$\bar{x}$	±SD	Min.	Max.	Q25	Me	Q75	*p*
Perception of quality of life	Study group (HD)	3.30	0.99	1.00	5.00	3.00	3.00	4.00	0.000
	Control group (K)	4.02	0.78	1.00	5.00	4.00	4.00	5.00	
Perception of health	Study group (HD)	2.43	0.99	1.00	5.00	2.00	3.00	3.00	0.000
	Control group (K)	4.08	0.99	1.00	4.00	4.00	4.00	5.00	

**Table 3:** Comparison of somatic, psychological, social, and environmental domains assessed by the WHOQOL-BREF quality-of-life questionnaire in the study groups, 0–100 scale (HD, K).

The Domains of Quality of Life Assessed by the WHOQOL-BREF Questionnaire		$\bar{x}$ (0–100)	±SD	Q25	Me	Q75	*p*
Somatic domain	Study group (HD)	56	23	43	54	64	0.0000
	Control group (K)	63	12	54	64	71	
Psychological domain	Study group (HD)	61	16	50	63	71	0.0000
	Control group (K)	72	10	67	75	79	
Social domain	Study group (HD)	65	42	50	67	75	0.0000
	Control group (K)	87	36	75	92	100	
Environmental domain	Study group (HD)	67	18	58	70	78	0.0000
	Control group (K)	82	14	75	84	91	

**Table 4:** The correlation between the examined domains of quality of life and age, gender, and level of education in the study group (HD) and control group (K).

Examined Issues		Study Group (HD)			Control Group (K)		
		N	R	*p*	N	R	*p*
Perception of quality of life	age	100	−0.22	0.0305	100	−0.09	0.3963
Perception of health	age	100	−0.10	0.3009	100	0.00	0.9810
Somatic domain	age	100	−0.20	0.0416	100	−0.10	0.3448
Psychological domain	age	100	−0.26	0.0091	100	−0.21	0.0387
Social domain	age	100	−0.34	0.0006	100	−0.24	0.0172
Environmental domain	age	100	−0.22	0.0255	100	0.02	0.8258
Perception of quality of life	sex (female)	100	−0.21	0.0368	100	0.08	0.4135
Perception of health	sex (female)	100	−0.27	0.0075	100	0.02	0.8653
Somatic domain	sex (female)	100	−0.27	0.0059	100	0.00	0.9611
Psychological domain	sex (female)	100	−0.26	0.0103	100	−0.03	0.7371
Social domain	sex (female)	100	−0.31	0.0018	100	−0.07	0.4790
Environmental domain	sex (female)	100	−0.29	0.0040	100	0.17	0.0862
Perception of quality of life	educational level	100	0.13	0.1811	100	0.25	0.0128
Perception of health	educational level	100	0.19	0.0618	100	0.11	0.2904
Somatic domain	educational level	100	0.09	0.3929	100	0.17	0.0915
Psychological domain	educational level	100	0.19	0.0522	100	0.19	0.0580
Social domain	educational level	100	0.18	0.0771	100	0.18	0.0670
Environmental domain	educational level	100	0.12	0.2439	100	0.13	0.2091

**Table 5:** Quality of life and stress in the study group (HD) and the control group (K).

Quality-of-Life Domains	Stress					
	Study Group (HD)			Control Group (K)		
	N	R	*p*	N	R	*p*
Perception of quality of life	100	0.09	0.3772	100	−0.23	0.0206
Perception of health	100	0.14	0.1598	100	−0.19	0.0551
Somatic domain	100	0.21	0.0320	100	−0.07	0.4977
Psychological domain	100	0.01	0.9273	100	−0.21	0.0350
Social domain	100	−0.07	0.5105	100	−0.05	0.6042
Environmental domain	100	0.07	0.5157	100	−0.01	0.9163

**Table 6:** Quality of life vs. periodontitis diagnosis according to Page and Eke [26], periodontal status and oral hygiene parameters (PD, CAL, GI, PI) in the study groups (HD, K).

Examined Issues		Study Group (HD)			Control Group (K)		
		*n*	R	*p*	*n*	R	*p*
Diagnosis of periodontitis according to Page and Eke	Perception of quality of life	100	−0.09	0.3935	100	−0.10	0.3253
	Perception of health	100	−0.15	0.1260	100	−0.08	0.4264
	Somatic domain	100	−0.15	0.1422	100	0.00	0.9824
	Psychological domain	100	−0.18	0.0705	100	0.00	0.9961
	Social domain	100	−0.23	0.0209	100	0.11	0.2749
	Environmental domain	100	0.02	0.8331	100	0.09	0.3511
Depth of periodontal pockets (PD)	Perception of quality of life	100	−0.07	0.5004	100	−0.18	0.0667
	Perception of health	100	−0.07	0.5004	100	−0.18	0.0673
	Somatic domain	100	−0.18	0.0813	100	−0.01	0.9149
	Psychological domain	100	−0.17	0.0841	100	−0.02	0.8288
	Social domain	100	−0.31	0.0018	100	0.03	0.7644
	Environmental domain	100	0.00	0.9865	100	−0.11	0.2631
Level of connective tissue attachment loss (CAL)	Perception of quality of life	100	−0.14	0.1592	100	−0.07	0.4627
	Perception of health	100	−0.09	0.3648	100	0.12	0.2325
	Somatic domain	100	−0.16	0.1170	100	−0.09	0.3572
	Psychological domain	100	−0.22	0.0298	100	0.06	0.5405
	Social domain	100	−0.27	0.0060	100	0.17	0.0885
	Environmental domain	100	−0.08	0.4156	100	0.14	0.1760
Mean value of Gingival Index according to Löe and Sillness	Perception of quality of life	100	0.01	0.9012	100	−0.18	0.0741
	Perception of health	100	−0.21	0.0322	100	0.00	0.9859
	Somatic domain	100	0.03	0.7382	100	−0.07	0.4923
	Psychological domain	100	−0.06	0.5527	100	−0.03	0.7507
	Social domain	100	−0.06	0.5806	100	−0.01	0.9133
	Environmental domain	100	0.07	0.5096	100	−0.10	0.3354
Plaque Index value according to Sillness and Löe	Perception of quality of life	100	−0.05	0.6230	100	−0.12	0.2450
	Perception of health	100	−0.12	0.2159	100	0.00	0.9831
	Somatic domain	100	−0.18	0.0709	100	0.05	0.6367
	Psychological domain	100	−0.20	0.0412	100	0.00	0.9915
	Social domain	100	−0.21	0.0351	100	−0.04	0.7199
	Environmental domain	100	−0.14	0.1713	100	−0.03	0.7431

**Table 7:** Quality of life vs. number of teeth retained in the oral cavity, tooth loss due to periodontal disease in the study groups (HD, K).

Examined Issues		Study Group (HD)			Control Group (K)		
		*n*	R	*p*	*n*	R	*p*
Number of teeth retained in the oral cavity	General quality of life	100	0.12	0.2205	100	0.19	0.0535
	General health quality	100	0.05	0.6158	100	0.12	0.2220
	Somatic domain	100	0.14	0.1605	100	0.13	0.1840
	Psychological domain	100	0.20	0.0490	100	0.19	0.0533
	Social domain	100	0.20	0.0467	100	0.21	0.0328
	Environmental domain	100	0.17	0.0979	100	0.02	0.8135
Tooth loss due to periodontal disease	General quality of life	100	−0.14	0.1590	100	−0.04	0.7165
	General health quality	100	−0.13	0.2130	100	−0.09	0.3708
	Somatic domain	100	−0.15	0.1285	100	−0.04	0.7132
	Psychological domain	100	−0.21	0.0394	100	0.04	0.7212
	Social domain	100	−0.24	0.0181	100	−0.05	0.6551
	Environmental domain	100	−0.04	0.7179	100	0.09	0.3704

**Table 8:** Quality of life and associated diseases in the study group (HD).

Study Group (HD)				
Examined Issues		N	R	*p*
Perception of quality of life	diabetes	100	−0.06	0.5408
Perception of health	diabetes	100	−0.11	0.2552
Somatic domain	diabetes	100	−0.10	0.3096
Psychological domain	diabetes	100	−0.28	0.0046
Social domain	diabetes	100	−0.28	0.0049
Environmental domain	diabetes	100	−0.08	0.4115
Perception of quality of life	Coronary-artery disease	100	−0.21	0.0388
Perception of health	Coronary-artery disease	100	−0.14	0.1671
Somatic domain	Coronary-artery disease	100	−0.22	0.0245
Psychological domain	Coronary-artery disease	100	−0.35	0.0003
Social domain	Coronary-artery disease	100	−0.14	0.1515
Environmental domain	Coronary-artery disease	100	−0.14	0.1786
Perception of quality of life	Rheumatoid arthritis	100	−0.24	0.0158
Perception of health	Rheumatoid arthritis	100	−0.14	0.1754
Somatic domain	Rheumatoid arthritis	100	−0.20	0.0513
Psychological domain	Rheumatoid arthritis	100	−0.12	0.2473
Social domain	Rheumatoid arthritis	100	−0.20	0.0507
Environmental domain	Rheumatoid arthritis	100	−0.22	0.0252
Perception of quality of life	Osteoporosis	100	−0.20	0.0495
Perception of health	Osteoporosis	100	−0.31	0.0018
Somatic domain	Osteoporosis	100	−0.18	0.0751
Psychological domain	Osteoporosis	100	−0.23	0.0196
Social domain	Osteoporosis	100	−0.36	0.0002
Environmental domain	Osteoporosis	100	−0.28	0.0041
Perception of quality of life	Oral mycosis	100	−0.29	0.0037
Perception of health	Oral mycosis	100	−0.15	0.1400
Somatic domain	Oral mycosis	100	−0.17	0.0912
Psychological domain	Oral mycosis	100	−0.19	0.0582
Social domain	Oral mycosis	100	−0.19	0.0594
Environmental domain	Oral mycosis	100	−0.15	0.1412

**Table 9:** Quality of life and limiting food intake due to periodontal disease in the study group (HD).

Quality-of-Life Domains	Restricting Food Intake Due to Periodontal Disease		
	Study Group (HD)		
	*n*	R	*p*
General Quality of life	100	−0.09	0.3772
General health quality	100	−0.14	0.1598
Somatic domain	100	−0.21	0.0320
Psychological domain	100	−0.01	0.9273
Social domain	100	−0.07	0.5105
Environmental domain	100	−0.07	0.5157

## Data Availability

Data available on request.
